# Modeling Fire Occurrence at the City Scale: A Comparison between Geographically Weighted Regression and Global Linear Regression

**DOI:** 10.3390/ijerph14040396

**Published:** 2017-04-08

**Authors:** Chao Song, Mei-Po Kwan, Jiping Zhu

**Affiliations:** 1 State Key Laboratory of Fire Science, University of Science and Technology of China, Hefei 230026, China; songch35@mail.ustc.edu.cn; 2 Department of Geography and Geographic Information Science, University of Illinois at Urbana-Champaign, 255 Computing Applications Building, MC-150, 605 E Springfield Ave., Champaign, IL 61820, USA; mpk654@gmail.com; 3 Department of Human Geography and Spatial Planning, Faculty of Geosciences, Utrecht University, P.O. Box 80125, 3508 TC Utrecht, The Netherlands

**Keywords:** GTWR, GWR, heterogeneity, space and time, global linear regression, fire risk

## Abstract

An increasing number of fires are occurring with the rapid development of cities, resulting in increased risk for human beings and the environment. This study compares geographically weighted regression-based models, including geographically weighted regression (GWR) and geographically and temporally weighted regression (GTWR), which integrates spatial and temporal effects and global linear regression models (LM) for modeling fire risk at the city scale. The results show that the road density and the spatial distribution of enterprises have the strongest influences on fire risk, which implies that we should focus on areas where roads and enterprises are densely clustered. In addition, locations with a large number of enterprises have fewer fire ignition records, probably because of strict management and prevention measures. A changing number of significant variables across space indicate that heterogeneity mainly exists in the northern and eastern rural and suburban areas of Hefei city, where human-related facilities or road construction are only clustered in the city sub-centers. GTWR can capture small changes in the spatiotemporal heterogeneity of the variables while GWR and LM cannot. An approach that integrates space and time enables us to better understand the dynamic changes in fire risk. Thus governments can use the results to manage fire safety at the city scale.

## 1. Introduction

Fire is a natural phenomenon that occurs worldwide in complex environments. Fire presents a great threat to people and the natural environment and scientists are aware of the necessity to manage fire risk. An example is the big fire that occurred in Chicago in 1871 when flying debris obscured the sky and Chicago was overcome by fire. To date, it is still a challenge for scientists to explain the occurrence and spread of natural fires, and it is even more difficult to predict fires. Fires have been studied in many regions around the world [1,2,3,4,5], such as in Spain [6,7] and many other countries [8]. However, few studies have examined fire risk at the city scale and its influence on the environment requires further studies. Worldwide, it is widely known that human activities play an important role in fire risk [9,10,11,12]. Dense urban population in urban areas and related hidden sources of hazards such as electrical installations and power lines may lead to the occurrence of fires as described in previous studies [13,14]. Places with a high population density such as markets and residential areas are especially vulnerable to high levels of fire risk. In addition, other factors such as road density, distance to water bodies, average temperature, precipitation, relative humidity, wind speed, slope, and aspect, which can be summarized as socioeconomic, climate and topographic predictors, were found to be associated with fire risk [7,13,15].

Several studies have adopted global linear regression models (LM) to study fire risk [15,16]. However, because of the relationship between fire risk and its influencing factors that may vary over space, which is referred to as spatial heterogeneity, LM, including ordinary least squares (OLS) models, may not be adequate for examining the spatially varying relationship between multiple predictors and fire occurrence. This limitation is mostly due to the constant coefficients in LM. Geographically weighted regression (GWR) has been widely used to take into account the spatial heterogeneity because of the unique characteristics of the model. GWR allows the regression coefficients to vary for individual locations, capturing the effects of non-stationarity and revealing variations in the importance of the variables across the study area. The use of GWR focuses particularly on data analysis and interpretation rather than prediction [8,16,17]. Aside from the popular spatially varied coefficient model, GWR was extended further and geographically and temporally weighted regression (GTWR) was developed to deal with both spatial and temporal non-stationarity [17]. GWR-based models are not just designed for improving model fitness; rather they facilitate the spatiotemporal exploration of natural phenomena.

GTWR integrates both temporal and spatial information in the weight matrices to capture spatial and temporal heterogeneity simultaneously [17,18]. The approach has been used in models of house prices and land use change [17,18,19]. The statistical performance of the GTWR is better than that of the GWR and the OLS in terms of goodness-of-fit. It is a well-known fact that fire risk is a phenomenon that changes over space and time [11,20,21,22]. The frequency of fire and its ignition locations show spatiotemporal dependence such as clustering, lagging and seasonal trends. Therefore, the existing fire models could be improved by incorporating temporal effects by integrating both spatial and temporal information in the weighting matrices [17,18,23]. Therefore, we aim to use GTWR to discover the potential rules for fire risk and to compare the approach with GWR and LM.

Before developing a GWR or GTWR model, the first task is to select variables and to fit an LM by adopting the OLS method for the purpose of comparison. The process of selecting variables is complex and different criteria or approaches may be used for different models, such as the Akaike information criterion (AIC), Bayesian information criterion (BIC), cross validation (CV), stepwise regression, and mean squared errors (MSE) reduction [15,24,25]. Further, an accuracy assessment of predictive spatial models needs to account for spatial autocorrelation. However, little attention has been paid to the influence arising from the presence of spatial autocorrelation in geospatial data and residuals, which may result in overfitting or underestimation [26]. By using spatial cross validation and bootstrap strategies, spatial prediction errors in the resampling-based accuracy assessment can be improved and the bias caused by residual spatial autocorrelation (RSA) can be corrected [26,27]. R statistical software and the packages “spgwr”, “sperrorest”, and “caret” have been used to calibrate the spatial cross validation (SCV) process and thus the resampling-based variable importance and prediction error across data folds can be achieved [26,28]. Therefore, it is necessary to first select variables by using SCV before using the variable in a further regression model.

This study compares geographically weighted regression-based models (including GWR and GTWR, which integrates spatial and temporal effect) and LM for modeling fire risk at the city scale. We use historical fire records and related datasets for Hefei city in China to undertake the comparative analysis. The study is divided into three tasks. First, SCV and CV are separately employed and compared in order to obtain the importance of the variables and then the relatively important predictors are selected after multicollinearity test. We also compare SCV and CV and identify the specific differences between them. Second, we adopt the selected variables from the previous step and fit the OLS model using the “caret” package in the R software. The significance level and the relative importance of the variables in the OLS model are quantified and the non-significant variables are removed. Third, we use the significant variables to fit a GWR model and visualize the local coefficients, the local significance of the coefficients and the residual distribution. GTWR is then employed and the fitness of the three models is summarized, along with a semivariogram analysis of residuals in different time periods. In this study, we adopted the original GTWR model created by Huang [18]. Therefore, the other improved GTWR models are not examined in the study.

The study shows that by using advanced GIS and spatial statistical methods along with detailed historical datasets of fire ignition, it is possible to build valid and meaningful models to explain fire risk. We can use them to improve the management of fire risk and safety in urban areas as well as in the natural environment. 

## 2. Materials and Methods

### 2.1. Study Area

**Figure 1:**
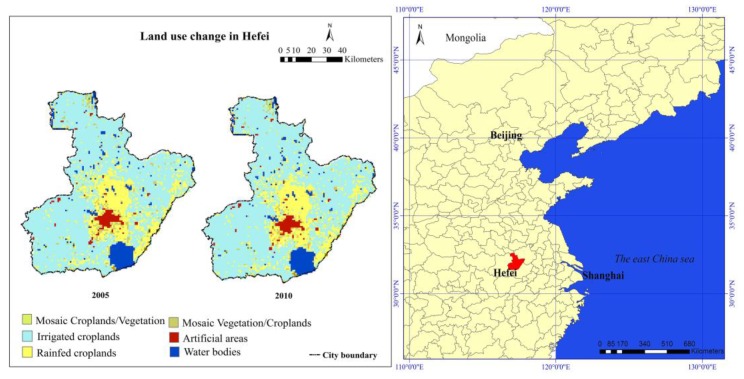
Land use in Hefei city in 2005 and 2010.

The study area is in Hefei city, which is located in the middle of Anhui Province in China. The city comprises a total area of around 7029 km^2^ and had a population of about five million prior to 2010. The land use in Hefei in 2005 and 2010 is shown in Figure 1. The data set with a spatial resolution of 300 m is provided by the Database of Global Change Parameters, Chinese Academy of Sciences [29].

**Figure 2:**
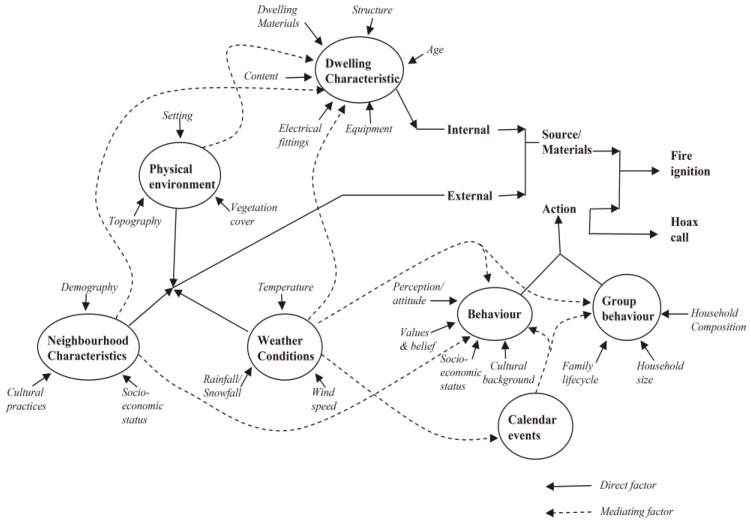
Conceptual model of fire risk [13]. (License No.: 4046910333494, permitted by Elsevier).

Although the study area is small relative to previous studies, there still exists considerable diversity in its socioeconomic, climate, topographic, and other attributes. In previous studies, researchers mainly adopted complex socioeconomic factors for fire occurrence and fire risk, which include population density, population structure, road density, slope, and other factors. These factors play important roles in fire occurrence, as shown in Figure 2, which is a conceptual summary of various fire risk factors and was developed by Corcoran et al. [13].

### 2.2. Dependent Variable and Preliminary Analysis for Infrastructure Fire Frequency

A dataset of fire records for the period of 2002–2010 in Hefei was obtained from the Fire Bureau in Anhui Province, China. The contained information includes the time of occurrence, event type, location, event damage, and fire-fighting time. A total of 12,629 historical infrastructure fire fighting records were extracted. The annual demographic and climate records including population, precipitation (Prec), relative humidity (Rehu), sunshine duration (Sun), temperature (Temp), GDP, and the average housing area of urban residents (UH) and rural residents (RH) in Hefei were provided by the statistical bureau of Anhui Province (http://www.ahtjj.gov.cn/tjj/web/index.jsp).

**Figure 3:**
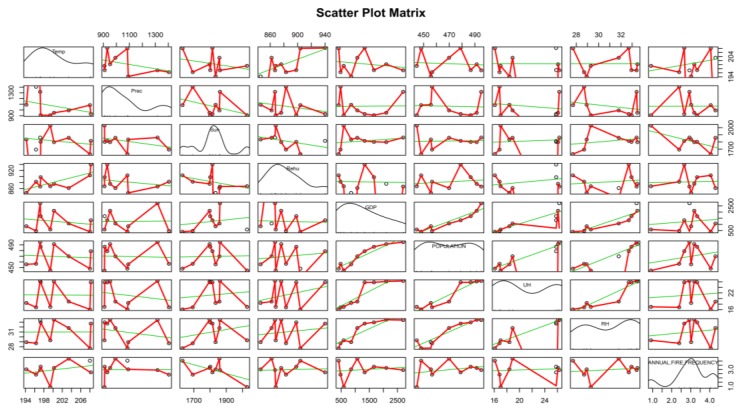
Scatter plot matrix for the internal relationships among annual predictors. Prec, Sun, Rehu, Sun, urban residents (UH), and rural residents (RH) are the abbreviations of precipitation, relative humidity, sunshine duration, temperature, average housing area of urban residents, and average housing area of rural residents respectively.

**Figure 4:**
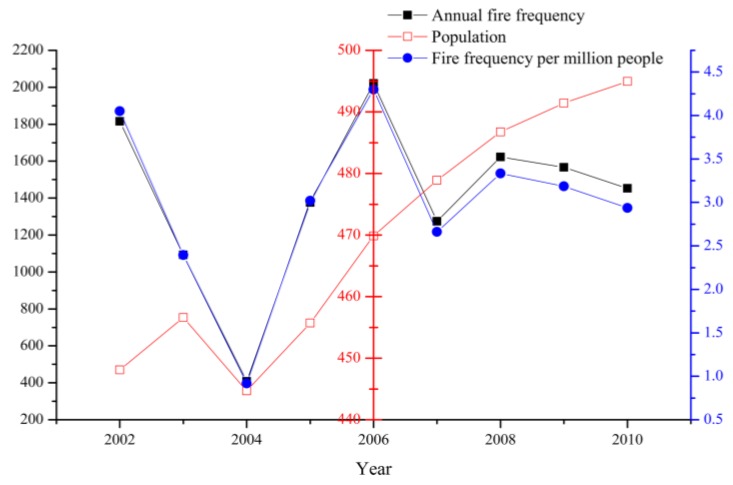
Annual summaries for fire records (2002–2010) and the relationship with population growth.

Moreover, we conducted a deeper analysis by using a scatter point matrix to study the internal relationships between the annual explanatory predictors and the per capita annual fire frequency. The results are shown in Figure 3, which indicates that almost all of the predictors have a certain degree of deviation. These predictors have a positive effect on fire frequency except sunshine duration. In addition, the correlations about the per capita annual fire frequency and the change in population are shown in Figure 4. It shows that the plot of per capita annual fire frequency has a very similar trend to the plot of annual fire frequency, which could be explained by the population growth and the fire risk caused by human activities.

Moreover, Figure 4 shows that in the latest years, fire frequency per capita has decreased more than fire frequency. Table 1 also shows that human-caused fires play an important role in explaining ignitions and places of interest (POI), and areas, where population is clustered, represent the main potential causes of fire risk, evidenced by the high proportion of fire occurring in those places, with the exception of fires associated with chemical industries, traffic, and electrical appliances.

**Figure 5:**
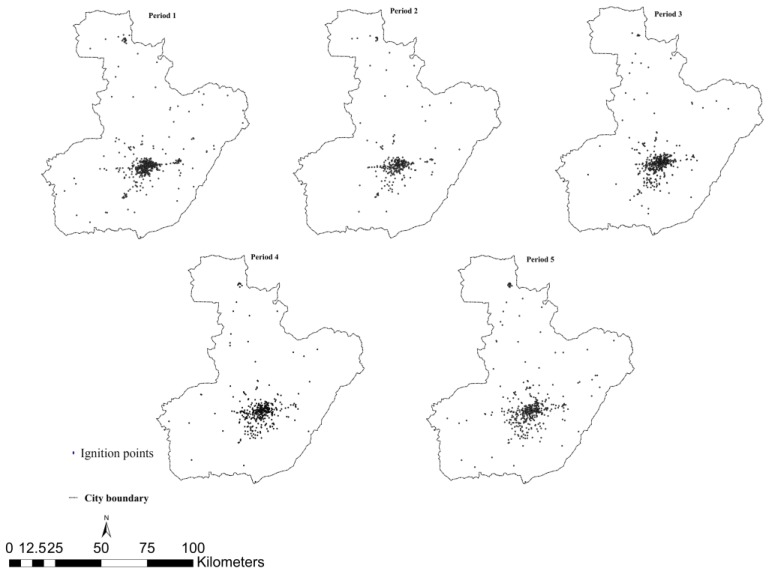
Ignition points between 2002 and 2010, divided into five time periods.

In order to make comparisons between GTWR and other models, we divided the entire time period of nine years into five temporal periods of nearly 22 months each. Thus, we could obtain the spatial distribution of the fire ignition points for different time periods. The spatial distribution of fire ignition points is shown in Figure 5 The response variable, called average fire density, was derived using the kernel density method, which turns discrete points in a study area into a continuous density surface to minimize the uncertainty and mistakes in the ignition records [15]. Fire density is defined in this study as the ignition frequency in one grid cell (occurrence number per period per km^2^).

A spatial resolution of 1 km × 1 km and a fixed bandwidth of 5 km were used as a rule of thumb after comparing several different bandwidth values (from 1 km to 10 km) [6]. Water bodies and similar land cover types where fire cannot occur were excluded from the analysis. The resulting base grid had 6985 cells covering the entire study area excluding water bodies. The centers of the pixels were used as the initial sample points. In order to mitigate the effect of spatial autocorrelation, rather than using all 6985 grids, 1000 sample points distributed randomly in space were selected for each time period, thus, 5000 sample points in total were used in the process of training the model. In addition, 500 samples dispersed spatially were used as validation samples across the five time periods.

### 2.3. Explanatory Variables: Selection and Pre-Processing

**Figure 6:**
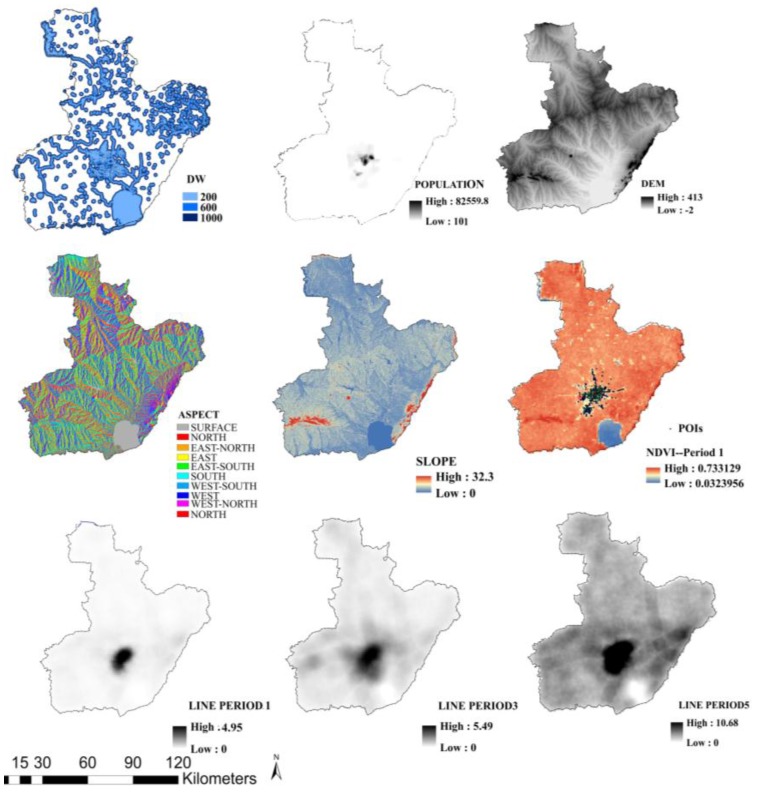
Illustration of explanatory variables.

A total of 25 explanatory variables covering a variety of socioeconomic attributes were extracted from the databases [13,30,31,32,33,34]. These variables consider the influence of socioeconomic conditions on fire occurrence, as well as the influence of climate and topographic conditions. These explanatory variables were derived from the previous literature and several new variables consisting of the spatial distribution of buildings were selected for the modeling process of infrastructure fire occurrence. These explanatory variables are shown in Table 2. Some variables, including static and dynamic variables, are illustrated in Figure 6. It should be noted that the uncertain geographic context problem (UGCoP) could affect the reallocation of fire risk spatiotemporally because of the dynamic change of socioeconomic attributes in urban areas [35]. This highlights the reason for the need of spatiotemporal models. All the explanatory variables were resampled to a resolution of 1 km × 1 km to achieve the same resolution as that of the fire density.

#### 2.3.1. Topography

Topographic features affect the spatial patterns, the composition, and the flammability of vegetation in addition to influencing local climatic conditions [7,8,31,37,38,39,40]. The elevation for Hefei city was obtained from the MODIS Global Digital Terrain Model (GDTM) 30-m resolution digital elevation model (DEM) dataset [41]. All topographic variables were resampled to 1 km by using the “resample” tool in ArcGIS 10.2. Areas of low elevation are more likely chosen for developing human settlements and thus capture the density of buildings. POSITION and SHADE were calculated based on the DEM dataset using the platform of the Computer Network Information Center at the Chinese Academy of Sciences, as shown in Table 2. Slope, aspect and terrain ruggedness index were calculated using the ArcGIS 10.2 (ESRI, Redlands, CA, USA) surface analysis tool and aspect was converted to a numeric variable with a range between −1 and 1 by using the aspect index based on the cosine function as shown in Equation (1) [42]:

(1) Aspect index=−cos(θ×2×π/360)

The proportion of the different topographic classes in each grid cell was then retrieved by using the “extract-multi-values-to-points” tool in ArcGIS 10.2. In total, six variables were obtained.

#### 2.3.2. Land Cover and NDVI

Different land cover types may reflect different sources that characterize the nature of fire [8,16]. As different land cover types reflect the potential area of human activities, it is important to pay attention to their distribution. Since previous studies have found a strong association between land cover types and fire occurrence [7,43], this study also considered the proportion of different land cover types that occurred at different time periods of the study (from 2002 to 2010). Land cover types whose proportion was too small (below 1%) or whose relevance to infrastructure fire was not high were excluded such as forest and grassland. The land cover types irrigated croplands, rain-fed croplands, mosaic cropland/vegetation, and artificial surfaces and associated areas were extracted from the land use map for the following analysis. The categories of land use type were converted into dummy variables, which facilitated the quantitative analysis as shown in Table 2. In order to diminish the effect of collinearity, LANDOTHER was excluded as the control group so it would not be in the model. The normalized difference vegetation index (NDVI) was used as the vegetation index for further analysis. Monthly MODND1D NDVI data was downloaded with a resolution of 500 m by using the platform of the Computer Network Information Center at the Chinese Academy of Sciences, as shown in Table 2. NDVI reflects the fuel greenness and the amount of actively growing vegetation in one grid cell [3]. The spatial distribution of NDVI for each time period was obtained by using “raster calculator” tool in ArcGIS 10.2, with which the layers of monthly NDVI were superimposed and averaged accordingly. Areas with a low value of NDVI likely indicate a higher cover of buildings than vegetation, suggesting where there is much greater likelihood of infrastructure fire occurrence.

#### 2.3.3. Temperature

The analysis includes three main climate variables: average surface temperature, maximum surface temperature, and minimum surface temperature (from 2002 to 2010). The temperature-related variables has the similar characteristics of spatial distribution in terms of the heat island effect, which can indicate the area where is the city center. The source of the original input dataset was the daily MOD11A1 data (version 5 with tile data) and we obtained the temperature variables by extracting the monthly MODIS synthetic products in China from the Computer Network Information Center at the Chinese Academy of Sciences (http://www.gscloud.cn). Afterwards, the average value of temperature in each time period was further processed by using the “raster calculator” tool in ArcGIS 10.2 thus we could get the value of temperature in each grid cell.

#### 2.3.4. Spatial Distribution of Population and Human Activities

Human activity is strongly related to fire occurrence [1,7,44,45,46]. The locations where human activities occur and where humans concentrate such as markets or hotels are also the locations of more frequent fires [13]. The spatial distribution of the population and the POIs such as business enterprises, educational facilities, residential areas, markets and hotels were included in this study. These types of POIs were seldom included in past studies of fire occurrence, which may lead to misleading results.

The geographic location and details about the POIs were obtained from EarthData Pacifica (Beijing) Co., Ltd. (http://www.geoknowledge.com.cn). A kernel density estimation with a fixed bandwidth was chosen to derive density surfaces for the other predictor variables including business enterprises, educational facilities, residential areas, markets, and hotels. The optimal fixed bandwidth was obtained by comparing a series of values from 5 km to 10 km, resulting in choosing 5 km for POIs except for hotels and educational facilities, where a bandwidth of 7 km was used.

Based on the method used for the Gridded Population of the World (version 4) for the estimation of human population density, we assigned population values to 30 arc-second (1 km) grid cells for 2000, 2005, and 2010 [36]. In detail, the population in period 2 can be obtained by interpolating the value of 2000 and 2005, and so on. The population density grids were derived by dividing the population count grids by the land area grids. The pixel values represent persons per square kilometer for the average distribution of the population in different temporal periods.

#### 2.3.5. Other Variables

In addition to the socioeconomic variables described above, the analysis also included other variables such as the distance from the ignition points to roads, the distance to water bodies, the distance to fire stations, and the line density of roads defined as road length per unit area. We chose these variables for modeling because they may affect the distribution of fire risk to some extent and even influence the losses caused by fire [47,48]. In addition, degree of freedom (DF) will definitely influence the final loss and it is a common sense, however, it will not affect the likelihood of fire occurrence. The values of these variables were obtained by integrating the “neighbor analysis”, “network analysis”, and the “line density” tools in ArcMap 10.2 (ESRI, Redlands, CA, USA) where the distance refers to Euclidean distance.

### 2.4. Models and Methods

#### 2.4.1. Data Preprocessing

Correlation coefficients that were too high (greater than 0.75) were used as the criterion to remove explanatory variables in order to mitigate the effects of multicollinearity among the explanatory variables [15]. In addition, data normalization and Box-Cox transformation were performed with the Z-score method by using SPSS software (IBM, North Castle, NY, USA) and R statistical software (R Development Core Team, Boston, MA, USA) in order to meet the basic assumption of normality for linear regression. 

#### 2.4.2. Variable Selection for LM

Five-thousand sample points were used for the training set and for the initial selection of variables and error estimation. As described in the introduction section, spatial cross validation (SCV) was employed and non-spatial cross-validation (CV) was calculated separately. The “sperrorest” and “errorest” packages in R software were used for this part of the analysis [26,49]. To be more specific, in order to obtain alternative estimates of the confidence intervals, a non-overlapping spatial k-means bootstrap approach was applied, which accounted for spatial autocorrelation. A 10-repeated-10-fold SCV/CV for LM adopting a k-means algorithm was applied, which meant that the whole study region was divided into 10 sub-regions and one fold was used as the testing set while the remaining nine folds were used as the training sets. 

After SCV and CV process, we removed the variables with a very small mean importance (less than 1.0 × 10^−4^), which meant that their contribution to fire risk could be safely neglected. We also obtained the kernel density estimation of the variable importance and the prediction error among the 100-fold data sets. 

#### 2.4.3. GWR and GTWR

The selected variables were included in the linear regression model using 10-repeated-10-fold CV in the “caret” package in the R software. The fitted model was further evaluated to assess outliers. Observations with a Cook’s distance greater than four times the mean may be classified as influential. The outliers were deleted and the variables which were not significant (with a *t*-test value of less than 1.96) were removed for the simplification and robustness of the model. The final predictors were then used to develop the GWR model by using an adaptive bandwidth searching approach. We chose a bisquare kernel function for building the spatial weight matrix and fitting the model by using “spgwr” package in R software. The results of the GWR model including the *t*-test values of the coefficients were interpolated visually by using ordinary kriging. We thus obtained the nonstationary spatial distribution characteristics for the contribution of the variables and revealed the varying significance levels across space.

In addition, the selected predictors were added to the modeling process of the GTWR which can uncover spatiotemporal heterogeneity. We adopted the original GTWR model created by Huang [18] and used a Gaussian kernel function to generate the spatiotemporal weight matrix. The program was developed in MATLAB R2014a (MathWorks, Natick, MA, USA), which provided the results including the coefficients and the *t*-test values for the included predictors. The results were then interpolated by using ordinary kriging to get a continuous surface of the coefficients and *t*-test values for the different time periods. Meanwhile, the goodness-of-fit was compared among the three models (LM, GWR, and GTWR). Based on the residual sum of squares (RSS) and the coefficient of determination (R-squared), we can compare the statistical performance of the GWR and GTWR models [18]. The variables might exhibit non-stationarity if the inter-quartile range (25% and 75% quartiles) of the GWR parameters is greater than ±1 standard deviation (SD) of the equivalent global OLS parameters [16]. This test was described in the following section.

In view of residual spatial autocorrelation (RSA), if no autocorrelation remained in the residuals of the regression models, the spatial pattern observed in the dependent variable could be explained by the spatial pattern observed in the predictors [15,27]. The residuals were obtained separately for the different periods. In order to analyze the explanatory power with regard to the spatial structure, semivariograms of the residuals with the function of distance were derived from the different models and these residuals were visualized with different colors to examine the heterogeneity and unstable performance of the models.

Furthermore, in order to investigate the potential regularity that influences the dynamic change of fire occurrence in different temporal periods, we standardized the change of the predictors for the grid cells of the training sets. Considering the characteristics of dynamic change for socio-economic and infrastructural predictors across temporal periods, we analyzed the correlation between the changes in fire density and the variable predictors, which can reflect the sensitivity and explain the reasons for changes in fire density over time. Moreover, the number of significant variables in the different spatial locations was evaluated for the five time periods. 

#### 2.4.4. Model Validation

Models were validated by using an independent dataset. Five-hundred sample points were used to compare the fitness among the three models and the RSS was calculated. The coefficient values of the predictors for the validation points were obtained by extraction from the continuous surface in ArcGIS 10.2. By comparing the models’ predictive accuracy for fire risk analysis, we will be able to choose a relatively robust model for fire prevention and better understand the dynamics of fire. 

## 3. Results

### 3.1. Results of the Variables Selection for the LM

**Figure 7:**
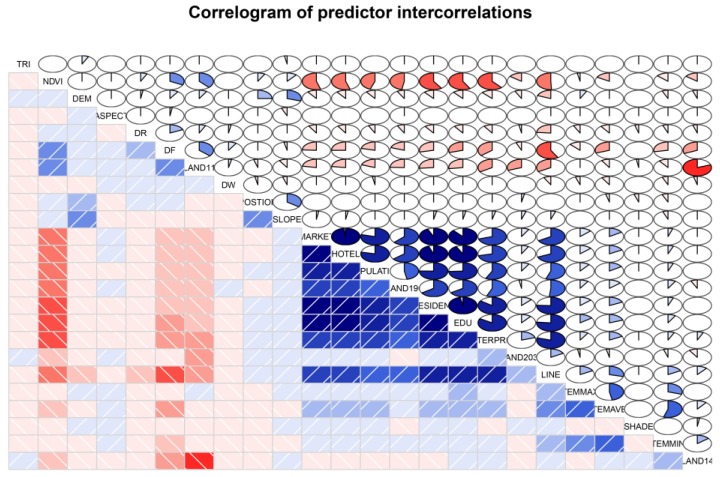
Correlogram of the explanatory variables. Red indicates a negative correlation and blue indicates a positive correlation. The larger the covered area in the fan diagram, the greater the absolute correlation value is.

All the variables were investigated and the fire density obtained by using the kernel density was converted by using a natural logarithm transformation. The variables ENTERPRISE, EDU, HOTEL, RESIDENT, and MARKET were converted by using a Box-Cox transformation when the value of *λ* was −0.5. All of the independent variables were then standardized. After the collinearity analysis that examined the correlations among the independent variables, LAND11, HOTEL, LANDOTHER and EDU were excluded from the modeling process. Further, we obtained the correlations among the predictor variables and visualized the results using the “corrgram” package in R studio (Figure 7). As shown in Figure 7, we reordered on the rows and columns of the matrix (made by principal component method) in order to have similar variables with related patterns together. We obtained the variance inflation factor (VIF) values of these variables except for the five dummy variables for the land use types. There was no VIF value greater than 5, indicating that there exists no multicollinearity among the variables. The remaining independent explanatory variables were included in the initial SCV and CV training processes.

**Figure 8:**
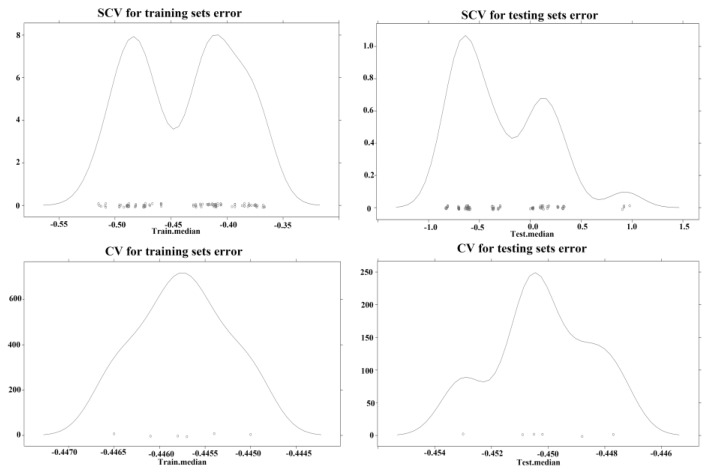
Kernel density plot for prediction error produced by SCV and CV. The small circles represent each data subset created from bootstrap.

Using global linear regression as the training model, estimates of the prediction error and variable importance were obtained as shown in Table 3. The resampling-based prediction error results across the 100-fold dataset are plotted in Figure 8, which indicates that the contribution to fire risk for each independent variable varies in the different sub training sets because the importance value is not constant. Furthermore, the difference between the absolute value of the prediction error is less for the SCV in the training set and the testing set than for the CV models. The figure also indicates that the prediction error in both training set and testing set for the SCV model is more dispersive while the prediction error is more gathered for the CV model, which may reflect that the prediction deviation is not normal for the CV. However, the mean importance for some variables changes considerably for different sub-regions, especially for LINE, RESIDENT, and NDVI, whose contribution to fire risk become negative in the random resampling process of the CV model. The results produced by the CV model may be unreliable because they are contrary to the common-sense knowledge that infrastructure fires - are often clustered in densely populated areas. 

As Table 3 shows, ENTERPRISE and LINE, which are closely related to human activities, were the most important variables influencing the value of fire risk. According to the results of the SCV model, POSITION, ASPECT, DR, DF, SHADE, TEMMAX, TRI, DW, and TEMMIN were removed because of their small importance values. The remaining 12 variables were used in the following LM training process. We infer that the distance to fire stations has little correlation with the probability of fire ignition, which means that the spatial distribution of the fire stations for improving response efficiency does not affect the occurrence of fires, though the fire stations should be located in areas with a high demand for service. In addition, some of the topographic predictors have little influence on fire risk such as SHADE, and those variables were deleted because of their low average value of variable importance.

### 3.2. Results of the LM and the GWR-Based Model

The remaining 12 variables were first used in the training process for the LM after the feature selection of the SCV. The regression results after the diagnosis of outliers in the LM results are shown in Table 4. LINE, ENTERPRISE, DEM, NDVI, LAND2030, TEMAVE, and SLOPE were significant and were included. Meanwhile, the adjusted R-squared in the LM was 0.2385 and the RSS was 3801.229. The degree of freedom (DF) in the LM was 4992. Both LINE and ENTERPRISE are important for the modeling of fire risk but their effect is opposite to each other. The higher the road density, the higher the density of fire is. However, the places where enterprises are clustered have a low fire density, because these locations are under a strict risk control management, leading to relatively scarce occurrence of fire risk.

Using the same data, the GWR and GTWR models were also tested and the results are reported in Table 5. “C” is the coefficient value of the LM, “Std. Error” is the standard error of the LM variable coefficients, ”-“ represents no value, and the bold text indicates the corresponding variable is significantly non-stationary or one of the quantile values is bigger than “C ± Std. Error”. The adaptive bandwidth was chosen as 0.374 and 0.242 for the GWR and GTWR models respectively, which meant the selected sample points were weighted for the local least squares process. The results indicated that local models based on the GWR had a better fit than the LM. The summary results show that the GTWR model has a better fit than the GWR model and the change in the local significance level occurs when the weight matrix is obtained from two to three dimensions. 

As seen in Table 5, all of the predictors except the intercept term are significant non-stationary variables in the GWR and GTWR models. However, the quantile range of predictors is different for the GTWR and GWR models. It is worth noting that the absolute values of the upper and lower limit of the coefficients are greater in the GTWR than in the GWR model. This reflects the need to consider the distribution of the selected predictors as a dynamic spatiotemporal parameter in predicting fire risk and the importance of considering the contribution of the time dimension to the fit of the model.

**Figure 9:**
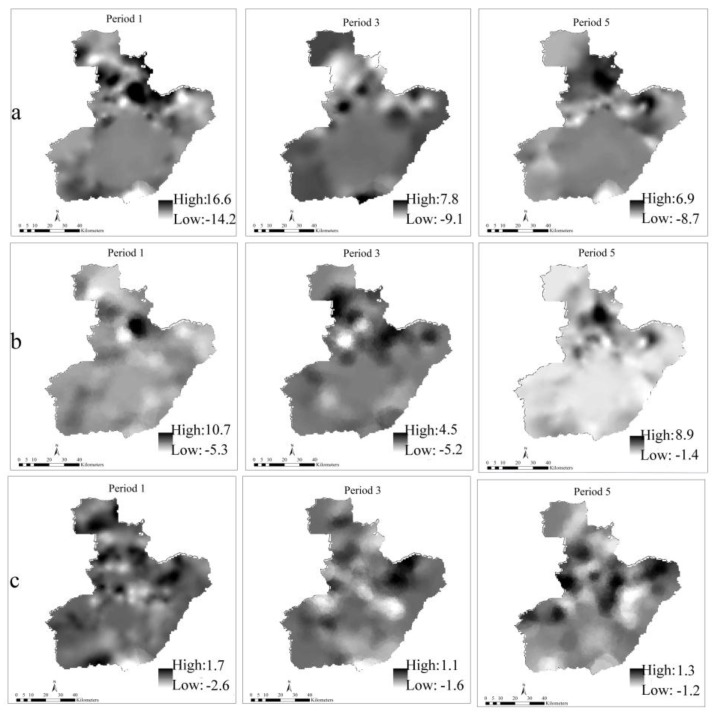
Distribution of the coefficients for GTWR in different periods at the resolution of 100 m. The letters (**a**–**c**) represent ENTERPRISE, LINE, and DEM respectively.

**Figure 10:**
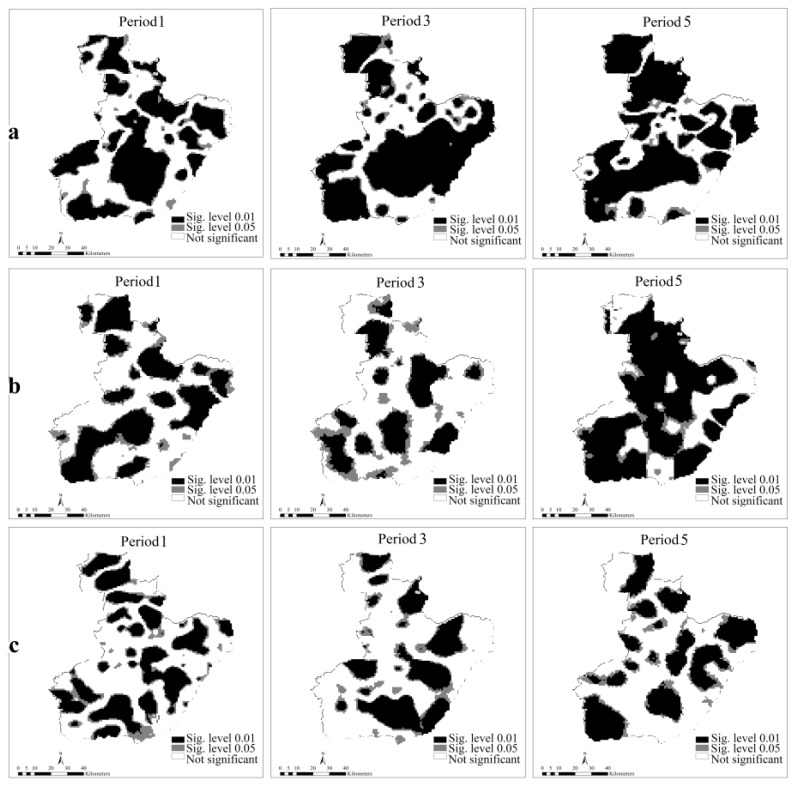
*t*-test values of the coefficients for GTWR in different periods at the resolution of 100 m. The letters (**a**–**c**) represent ENTERPRISE, LINE, and DEM respectively. The darker of the color, the more significant the coefficient is. White means the variable is not significant within that region, black indicates the variable is at the significance level of 0.01, and dark gray indicates a significance level of 0.05 (*t*-test value is 2.58 and 1.96 respectively).

Additional details about the distribution and varying significance of the coefficients for the GTWR model across space-time are shown in Figure 9 and Figure 10. By taking the periods 1, 3, and 5 as an example, the varying distribution of the coefficients and the correspondent *t*-test values (at the significance levels of 0.01 and 0.05) are somewhat different for the three periods. We created graphs of ENTERPRISE, LINE, and DEM (a, b, and c) as an example because of limited page space. For each predictor, the coefficient and the *t*-test value change over time. In addition, heterogeneity exists both in space and time as the varied parameter values in Figure 9 and Figure 10 indicate. 

The dark areas of ENTERPRISE and LINE indicate a positive effect mainly in some northern and northeastern regions of the city and this may uncover strong spatial variability. Therefore, we can determine the areas with different significance level, allowing us to make dynamic decisions to prevent fire occurrence.

### 3.3. Test of Spatial Autocorrelation for Residuals

**Figure 11:**
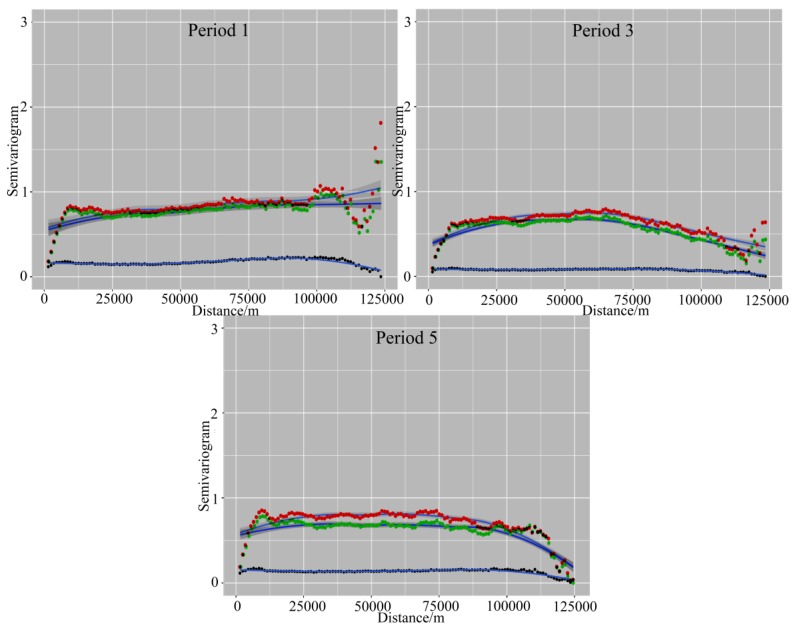
Semivariograms of the three models (LM in red, GWR in green and GTWR in black; the blue curve is the fitting plot).

**Figure 12:**
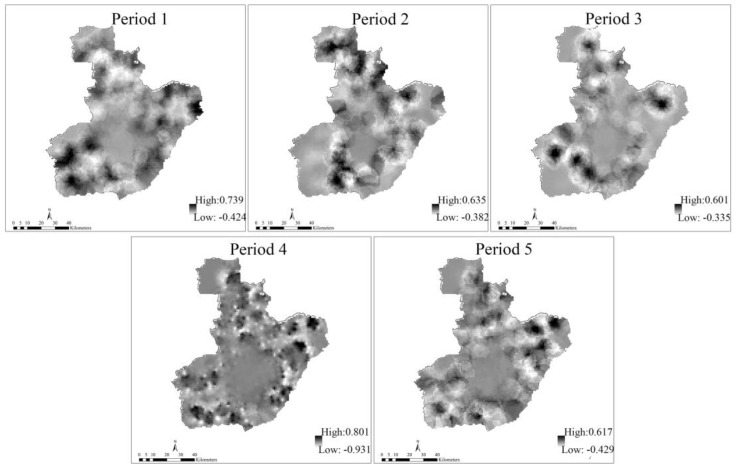
Spatial distribution of the residuals for GTWR.

The spatial distribution of the residuals was tested by using semivariograms and taking period 1, 3 and 5 as an illustration (Figure 11). As for GWR and LM, the semivariograms indicate a spatial autocorrelation for the residuals of the dependent variable with a spatial lag of up to nearly 12 km; the semivariance remains rather steady up to 100 km, beyond which it increases again to some extent. This is good proof that the various spatial clustering patterns change at different spatial scales. The GTWR model showed lower values of semivariance than the other two models, and exhibited a flat semivariance line for the entire distance, as indicated by the fit curve in Figure 11. The results show a strong ability of the GTWR model for explaining the spatial structure. The distribution of the residuals for all time periods is shown in Figure 12, which indicates that the residuals in the GTWR model are mostly generated outside the center of the city. In addition, the spatial shape and scope of the distribution of the residuals are different for the five time periods.

### 3.4. Assessment of Independent Validation for the Models

An independent validation was applied initially to the 500 sample points across the different time periods. Afterward, only 491 of these 500 sample points were used in the validation process after eliminating the points without effective values. The final GWR and GTWR models and their related predictor coefficient values were extracted and the RSS were obtained as shown in Table 6. The GTWR model proved more robust in the independent validation because it’s the model that has the lowest RSS value, which indicates that for our dataset, the GTWR model is assumed the best prediction model. The results for the RSS are nearly identical for the GWR (571.516) as for the LM (570.207).

Although statistical performance varies between the different time periods and considering the differences in the fitting mechanism of the GTWR and GWR models, we may infer that the GTWR model performs best not only in the training sets but also in the independent validation sets. Previous research has shown that GTWR was statistically the best among LM, GWR, and GTWR [17,18] and our results provide further evidence of this.

### 3.5. Heterogeneity of the Variable Significance Level

**Figure 13:**
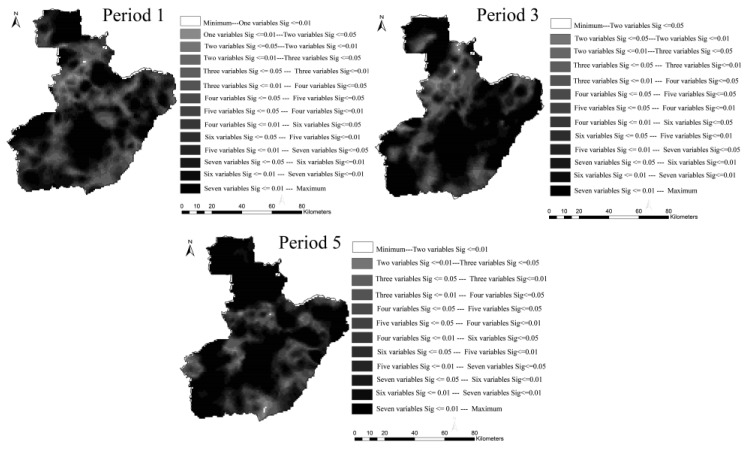
Number of variables with different significance levels for GTWR.

The number of variables which are significant at different levels is unevenly distributed in space and time. As shown in Figure 13, we observed that for the GTWR model in different time periods, the number of variables with different significance levels (0.01 and 0.05) varied across the entire city.

Meanwhile, the figure indicates that all seven variables are significant in the center area, which has the densest population while the number of significant variables changes hierarchically in some northern and eastern regions. The results show that the heterogeneity mainly exists in rural areas where human-related facilities or road construction are only clustered in the sub-centers. GTWR can detect imperceptible changes and this finding illustrates the advantage of GTWR when compared to GWR and LM.

### 3.6. Spatiotemporal Changes in Fire Density

**Figure 14:**
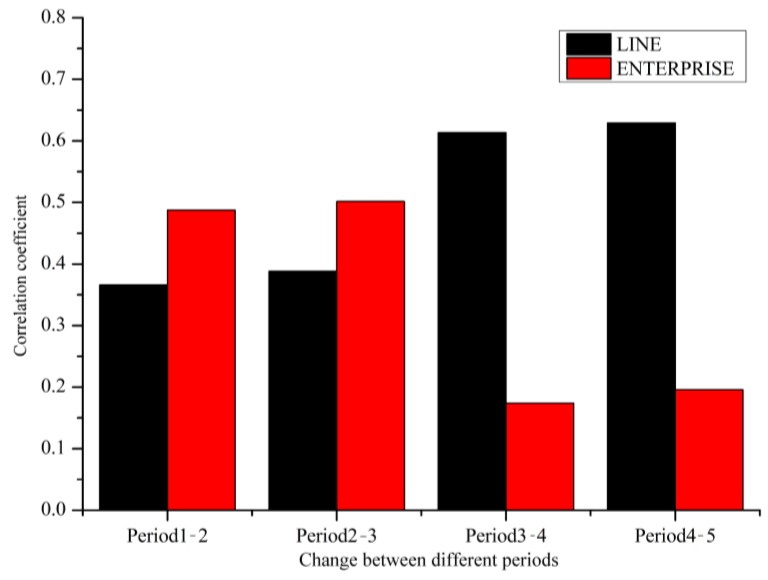
Histogram plot of the correlation between the change of predictors and the change of fire density.

We studied the causes of the spatiotemporal changes in fire occurrence at the city scale. The seven predictors chosen by the models were not constant all the time and LINE and ENTERPRISE were the most important variables. Therefore, the changes in value of the grid cells of LINE and ENTERPRISE were calculated by subtraction from period 1 to period 2, etc., for all periods. The changes in value were standardized and thus a correlation coefficient between the change of variable and the change of fire density was obtained (Figure 14). As indicated in Figure 14, although ENTERPRISE is the most important variable in the GTWR model, the variable which influences the change in fire density temporally is varying. ENTERPRISE influences the change in fire density more than LINE prior to period 2, close to the year of 2005. LINE plays a more important role than ENTERPRISE after period 3, which can be probably explained by the expansion of the urban region and the changes in the shape of the city. An increasing number of buildings and infrastructure have been constructed in the newly built area, called the city sub-center, and a dense road network and related supporting facilities have been developed. The improvement in access by the population will indirectly contribute to the growth of fire occurrence. On the other hand, by implementing technology to control fire risk in enterprises, the frequency of fire is less than before. Moreover, with the increase in land costs in urban areas, an increasing number of enterprises have moved to the suburbs and as the population will also increase in these areas, more sub-centers of fire risk will develop. 

## 4. Conclusions

In this study, we first performed a spatial cross validation for a linear regression model and compared its results with a stochastic cross validation. The contribution to fire risk by variables varied in different sub training sets and we infer that this kind of nonstationary situation also existed across space and that SCV could reduce the prediction error. The results also showed that the variables LINE and ENTERPRISE were the most important variables for modeling fire risk, although their effects on fire risk were opposite to each other. Further, the results indicated that road density and the population distribution had the most positive influence on fire risk, which implies that we should pay more attention to locations where roads and people are densely clustered. The results also showed that areas with a large number of enterprises had fewer fire ignition records, probably because of strict fire management and prevention measures. Infrastructure fire risk was commonly clustered in areas with dense population and increased human activities, which was in line with the common-sense knowledge.

The study compared LM, GWR, and GTWR by using the variables with a high mean importance value, which were used in the modeling process for fire risk at the city scale. LINE, ENTERPRISE, DEM, SLOPE, LAND2030, and TEMAVE which were all significant were employed in the LM first. The results showed that constant coefficient models like LM did not predict fire risk accurately and could not reveal the spatiotemporal heterogeneity. The statistical results highlighted the weakness of the LM considering the low R-squared value.

With regard to GWR-based methods, the statistical performance improved when compared to OLS and the GTWR was the best model. The R-squared values were 0.2385, 0.2837, and 0.8705 for OLS, GWR and GTWR respectively as shown in Table 5. More details on the distribution and varying significance of the coefficients for GTWR across space-time were illustrated and the varying distribution of the coefficients together with the correspondent *t*-test values (at the significance level of 0.01 and 0.05) changed to some extent for the different periods.

With regard to the spatial distribution of the residuals, the semivariograms indicated spatial autocorrelation for GWR and LM up to a 12-km lag, with a relatively steady semivariance up to 100 km, beyond which it increased again to some extent. This is good proof that the various spatial clustering patterns changed at different spatial scales. The GTWR model showed lower values of semivariance than the GWR and the LM, as well as a flat semivariance line for the entire distance. The results showed the strong ability of GTWR to explain the spatial structure.

For the validation process, GTWR proved more robust because the model had the lowest RSS value, which indicates that for our dataset, the GTWR is the best of the fitted regression models. In addition, a deeper exploration of the GWR revealed the heterogeneity to some extent, although the gap between GWR and GTWR was significant. The results indicate that all seven selected variables are significant in the center areas which have the densest population while the number of significant variables changed hierarchically in some northern and eastern regions. The results show that the heterogeneity mainly exists in suburban and rural areas where human-related facilities or road construction are only clustered in some sub-centers of a city. GTWR can capture small changes while GWR cannot. This finding further illustrated the advantage of GTWR when compared to GWR and LM.

In addition, an in-depth analysis of the relationship between the change in predictors and the change in fire density was conducted. The results show that the variable that influences the change in fire density temporally is varying. This can be probably explained by the expansion of the urban region and the changes in the shape of the city. In addition, an increasing number of buildings and infrastructures have been constructed in the newly built area and the improvement in access by the population will indirectly contribute to the growth of fire occurrence. On the other hand, by implementing technology to control the fire risk in enterprises, the frequency of fire is less than before. Moreover, more sub-centers of fire risk will develop.

The findings in this paper reveal the advantages of using GTWR for explaining fire risk spatiotemporally. This approach, which integrates space and time, enables us to understand the dynamic change in fire risk. Further, we can also make accurate predictions by using the variables that have a high correlation with fire risk in city areas. Therefore, we can determine the areas with different significance levels, allowing us to make dynamic decisions to prevent fire occurrence. An additional finding of this study was that the calculation of the bandwidth used in GTWR will also influence the results and this aspect should be studied further in the future. 

## Figures and Tables

**Table 1:** Statistical summary for fire categories.

Category	Number	Proportion
Population-clustered places (hotel, school, market, etc.)	2993	0.502941
Other	967	0.162494
Traffic-related	938	0.157621
Important buildings (warehouse, gas stations, etc.)	566	0.09511
Electricity	256	0.043018
High-rise buildings	109	0.018316
Chemical industries	88	0.014787
Underground buildings	34	0.005713

**Table 2:** Candidate explanatory variables.

Variable Name	Code	Data Source	Resolution/Unit
Elevation	DEM	The data set is provided by Geospatial Data Cloud site, Computer Network Information Center, Chinese Academy of Sciences (http://www.gscloud.cn)	30 m
Slope	SLOPE	Calculated by ArcGis 10.2 surface analysis tool	30 m
Aspect	ASPECT	The same as SLOPE	30 m
Topographic Position Index	POSITION	The same as DEM	30 m
Terrain Ruggedness Index	TRI	The same as DEM	30 m
Shaded relief	SHADE	The same as SLOPE	30 m
Normalized Difference Vegetation Index	NDVI	The same as DEM	500 m
Yearly average maximum surface temperature	TEMMAX	The same as DEM	1 km
Yearly average minimum surface temperature	TEMMIN	The same as DEM	1 km
Yearly average mean surface temperature	TEMAVE	The same as DEM	1 km
Population	POPULATION	GPWv4, NASA Socioeconomic Data and Applications Center (SEDAC) [36]	1 km
Line density of roads	LINE	Product Specification of Earth Data Pacifica (Beijing) Co., Ltd. (http://www.geoknowledge.com.cn)	1 km
Kernel density of residential points	RESIDENT	The same as LINE	1 km
Kernel density of market points	MARKET	The same as LINE	1 km
Kernel density of hotel points	HOTEL	The same as LINE	1 km
Kernel density of schools, universities, etc.	EDU	The same as LINE	1 km
Kernel density of enterprise points	ENTERPRISE	The same as LINE	1 km
Value of 11 for land cover- Post-flooding or irrigated croplands	LAND11	The data set is provided by Database of Global Change Parameters, Chinese Academy of Sciences. (http://globalchange.nsdc.cn)	300 m
Value of 14 for land cover- Rainfed croplands	LAND14	The same as LAND11	300 m
Value of 20 and 30 for land cover- Mosaic cropland/vegetation	LAND2030	The same as LAND11	300 m
Value of 190 for land cover- Artificial surfaces and associated areas	LAND190	The same as LAND11	300 m
The other values of land cover	LANDOTHER	The same as LAND11	300 m
Distance to water bodies	DW	The same as LINE and calculated by ArcMap 10.2 spatial analysis toolbox	m
Distance to fire stations	DF	The same as DW	m
Distance to roads	DR	The same as DW	m

DEM: digital elevation model.

**Table 3:** Results of SCV and CV for linear regression.

Indicators	LM	
	SCV	CV
Mean of Train.error	−0.371	−0.450
Mean of Test.error	−0.426	−0.446
Mean Imp of LINE	6.30 × 10^−^^3^	*−1.79 × 10^−3^*
Mean Imp of POPULATION	6.03 × 10^−^^3^	1.11 × 10^−^^4^
Mean Imp of LAND2030	2.55 × 10^−^^3^	3.39 × 10^−^^3^
Mean Imp of RESIDENT	2.10 × 10^−^^3^	*−1.37 × 10^−4^*
Mean Imp of LAND190	1.39 × 10^−^^3^	3.85 × 10^−^^3^
Mean Imp of LAND14	1.07 × 10^−^^3^	*−6.83 × 10^−3^*
Mean Imp of POSITION	5.72 × 10^−^^4^	*−9.51 × 10^−4^*
Mean Imp of ASPECT	4.11 × 10^−^^4^	1.14 × 10^−^^3^
Mean Imp of DR	3.60 × 10^−^^4^	4.15 × 10^−^^4^
Mean Imp of SHADE	1.80 × 10^−^^4^	*−2.17 × 10^−4^*
Mean Imp of TEMMAX	1.60 × 10^−^^5^	*−1.65 × 10^−3^*
Mean Imp of TRI	*−8.20 × 10^−4^*	*−5.48 × 10^−4^*
Mean Imp of DW	*−9.61 × 10^−4^*	2.61 × 10^−^^4^
Mean Imp of DF	*−1.10 × 10^−3^*	3.85 × 10^−^^2^
Mean Imp of SLOPE	*−1.65 × 10^−3^*	6.41 × 10^−^^3^
Mean Imp of TEMAVE	*−1.87 × 10^−3^*	6.65 × 10^−^^3^
Mean Imp of TEMMIN	*−6.32 × 10^−3^*	1.79 × 10^−^^2^
Mean Imp of MARKET	*−9.41 × 10^−3^*	1.22 × 10^−^^3^
Mean Imp of DEM	*−9.81 × 10^−3^*	*−7.00 × 10^−4^*
Mean Imp of NDVI	*−1.01 × 10^−2^*	1.25 × 10^−^^2^
Mean Imp of ENTERPRISE	*−4.53 × 10^−2^*	*−3.43 × 10^−3^*

“Imp” means the mean importance of variables. The underlined and italic values represent a negative effect towards the response variable while normal text represents a positive effect.

**Table 4:** Statistical summary of the LM.

Explanatory Variables	Coefficient (C)	Std. Error	*t* Value	Pr (>|t|)	
Intercept	0.00001	0.01234	−0.001	0.9995	
LINE	0.12850	0.01465	8.771	<2.0 × 10^−^^16^	***
ENTERPRISE	−0.32200	0.01581	−20.364	<2.0 × 10^−^^16^	***
DEM	−0.07834	0.01360	−5.759	0.000001	***
NDVI	−0.07091	0.01416	−5.007	<2.0 × 10^−^^16^	***
LAND2030	−0.02292	0.01262	−1.816	0.0694	†
TEMAVE	0.07167	0.01326	5.404	0.000001	***
SLOPE	−0.02595	0.01324	−1.960	0.0500	†

“***” means the significance is at the level of 0.001 and “†” means the significance is at the level of 0.1.

**Table 5:** Statistical summary of the GWR and GTWR models.

Explanatory Variables	GWR		GTWR	
	Quantile (25%, 75%)	C ± Std. Error	Quantile (25%, 75%)	C ± Std. Error
Intercept	**(−0.0100, 0.2175)**	(−0.0123, 0.0123)	-	-
LINE	**(0.0862, 0.5346)**	(0.1139, 0.1432)	**(−0.0070, 1.4744)**	(0.1139, 0.1432)
ENTERPRISE	**(−0.3433, −0.2143)**	(−0.3378, −0.3062)	**(−1.7656, 0.4415)**	(−0.3378, −0.3062)
DEM	**(−0.1752, −0.0248)**	(−0.0919, −0.0647)	**(−0.2915, 0.2071)**	(−0.0919, −0.0647)
NDVI	**(−0.1225, 0.0117)**	(−0.0851, −0.0568)	(**−0.1230**, **0.1369**)	(−0.0851, −0.0568)
LAND2030	**(−0.0462, −0.0013)**	(−0.0355, −0.0103)	(**−0.1097**, **0.1554**)	(−0.0355, −0.0103)
TEMAVE	**(0.0308, 0.0949)**	(−0.0584, 0.0849)	(**−0.1026**, **0.3715**)	(0.0584,0.0849)
SLOPE	**(−0.0422, 0.0092)**	(−0.0392, −0.0127)	(**−0.1376, 0.0880**)	(−0.0392, −0.0127)
R squared	0.2837		0.8705	
RSS	3403.22		646.52	
RSS improvement	GWR vs. LM: −398.01 GTWR vs. LM: −3154.71 GTWR vs. GWR: −2756.70			

“C” is the coefficient value of LM. “Std. Error” is the standard error of LM variable coefficients; “-” represents no value and the bold texts indicate that the corresponding variables are significantly non-stationary.

**Table 6:** Statistical comparison among the three models (accounting for the RSS).

Time Period	Model		
	LM	GWR	GTWR
1	567.517	567.919	507.709
2	573.082	574.668	506.101
3	569.504	570.852	507.263
4	575.697	572.586	508.224
5	565.234	571.556	510.529
RSS Average value	570.207	571.516	507.965
